# COVID-19 Preventive Measures in Northern California Jails: Perceived Deficiencies, Barriers, and Unintended Harms

**DOI:** 10.3389/fpubh.2022.854343

**Published:** 2022-06-14

**Authors:** Yiran E. Liu, Christopher LeBoa, Marcela Rodriguez, Beruk Sherif, Chrisele Trinidad, Michael del Rosario, Sophie Allen, Christine Clifford, Jennifer Redding, Wei-ting Chen, Lisa G. Rosas, Carlos Morales, Alexander Chyorny, Jason R. Andrews

**Affiliations:** ^1^Division of Infectious Diseases and Geographic Medicine, Department of Medicine, Stanford, CA, United States; ^2^Cancer Biology Graduate Program, Stanford University School of Medicine, Stanford, CA, United States; ^3^Stanford Center for Clinical Research, Stanford University School of Medicine, Stanford, CA, United States; ^4^Division of Correctional Health Services, San Mateo County Health, Redwood City, CA, United States; ^5^Stanford Law School, Stanford, CA, United States; ^6^Department of Sociology, Stanford School of Humanities and Sciences, Stanford, CA, United States; ^7^Silicon Valley De-Bug, San Jose, CA, United States; ^8^Santa Clara County Office of the Public Defender, San Jose, CA, United States; ^9^Office of Community Engagement, Stanford University School of Medicine, Stanford, CA, United States; ^10^Division of Custody Health, Department of Medicine, Santa Clara Valley Health and Hospital System, San Jose, CA, United States

**Keywords:** COVID-19, incarceration, jails, infection control, stakeholder engagement, community-based research, seroprevalence, mental health

## Abstract

**Background:**

Carceral facilities are high-risk settings for COVID-19 transmission. Little is known about the hidden burden of infection or practical barriers to infection control in these settings, especially in jails. There is also limited research on the mental health impacts of the pandemic among people living and working in carceral facilities.

**Methods:**

Between July 8, 2020 and April 30, 2021, we performed SARS-CoV-2 rapid antibody testing and administered a questionnaire among residents and staff of four Northern California jails. We utilized multivariable logistic regression, adjusting for demographic and carceral characteristics, to analyze factors associated with prior infection, including perceived likelihood of prior infection and access to new masks. We additionally assessed the implementation of, perceptions toward, and impacts of COVID-19 policies in practice. We engaged stakeholder representatives, including incarcerated individuals, to guide study design, procedures, and results interpretation.

**Results:**

We enrolled 788 jail residents and 380 jail staff. Nearly half of residents and two-thirds of staff who were antibody-positive had not previously tested positive for COVID-19. Among residents without a prior COVID-19 diagnosis, antibody positivity was significantly associated with perceived likelihood of prior infection (adjusted OR = 8.9; 95% CI, 3.6–22.0). Residents who had flu-like illness in jail cited inadequate responses to reported illness and deterrents to symptom reporting, including fears of medical isolation and perceptions of medical neglect. Residents also disclosed deficient access to face masks, which was associated with antibody positivity (adjusted OR = 13.8, 95% CI, 1.8–107.0). Worsened mental health was pervasive among residents, attributed not only to fear of COVID-19 and unsanitary jail conditions but also to intensified isolation and deprivation due to pandemic restrictions on in-person visitation, programs, and recreation time.

**Conclusion:**

Carceral settings present significant challenges to maintaining infection control and human rights. Custody officials should work diligently to transform the conditions of medical isolation, which could mitigate deterrents to symptom reporting. Furthermore, they should minimize use of restrictive measures like lockdowns and suspension of visitation that exacerbate the mental health harms of incarceration. Instead, custody officials should ensure comprehensive implementation of other preventive strategies like masking, testing, and vaccination, in conjunction with multisector efforts to advance decarceration.

## Introduction

Prisons, jails, and detention centers present numerous challenges to public health, especially during the COVID-19 pandemic (1–3). Physical distancing is difficult in congregate settings, and people residing in carceral facilities may experience inadequate access to personal protective equipment (PPE), sanitation, and medical care (4–8). Furthermore, restrictive strategies for infection control in carceral settings such as facility lockdowns and suspension of visitation and programming may be especially harmful to well-being in an already isolated population with high rates of pre-existing mental illnesses and medical conditions (4, 9–14).

Recognizing these challenges, many public health practitioners and researchers have called for large-scale decarceration (7, 15–19); however, most policies enabling decarceration have been short-lived (20). For the millions of incarcerated individuals and staff who continue to be exposed daily to high-risk carceral settings, major knowledge gaps persist that preclude evidence-based improvements to infection prevention and control in carceral facilities (21–23). First, the hidden burden of COVID-19 in carceral settings remains unclear. While confirmed case counts in carceral facilities are already alarmingly high, they are likely underestimated due to inadequate testing, asymptomatic transmission, and population turnover (24–28). Antibody testing, which can identify prior infection by detecting antibodies to SARS-CoV-2 in the blood, is one strategy to assess the extent of undetected infection in a population and to determine factors associated with risk of prior infection (29, 30). However, to our knowledge few studies–none in the U.S.–have employed antibody testing in a carceral setting (31–35).

We also have a poor understanding of how COVID-19 preventive measures in prisons and jails fared in practice (23). Although vaccination has become an effective strategy (36), its utility is limited by insufficient vaccine uptake among incarcerated individuals and staff (37–39) and new viral variants that may evade existing immunity (40–42). Therefore, it is vital to maintain other preventive strategies such as masking and testing. However, little is known about the de facto implementation of such measures in carceral settings. While media outlets, advocacy groups, and human rights organizations have documented deficiencies in practice (3, 8, 28, 43, 44), the extent and direct consequences of these deficiencies remain unclear. Relatedly, there has been little research on the perceptions of incarcerated individuals or staff toward COVID-19 policies, or on the unintended impacts of such policies, both of which can influence acceptability and effectiveness (45, 46). For instance, medical isolation and quarantine may be damaging for mental health in a carceral setting and may stoke fears that disincentivize testing uptake (11, 43, 46, 47).

These knowledge gaps are especially dire for jails, which generally have worse data transparency than prisons despite their potentially outsized role in COVID-19 transmission (27, 48–50). Whereas in prisons, all residents are serving sentences of years or more, in jails most residents are legally innocent and being held pre-trial, with the remainder serving shorter sentences (51). As a result, jails have higher rates of population turnover than prisons, with an estimated 4.9 million people passing through jails each year (52). This phenomenon of “jail churn,” on top of the daily commutes of hundreds of thousands of jail staff (53), may compound the risk of outbreaks and infection spillover into outside communities (17, 18).

Against this backdrop, we conducted a cross-sectional study across four county jails in Northern California to shed light on three understudied but important topics: (1) the extent of, and potential contributors to, undetected COVID-19 infection in carceral settings; (2) the implementation of preventive measures in practice, and perceptions toward them among incarcerated individuals and staff; and (3) the effects of restrictive COVID-19 policies on the health and well-being of incarcerated individuals and staff. To do this, we performed SARS-CoV-2 antibody testing and administered self-report questionnaires to people living and working in these four jails. In order to ensure the study's relevance and sensitivity for stakeholder populations, we engaged a community advisory board (CAB) consisting of incarcerated individuals, local and national advocates for criminal justice reform, a public defender, and custody health representatives to inform study design, procedures, and results interpretation. With guidance from the CAB, we hypothesized that flaws in the operationalization of preventive measures such as the response to reported illness or the provision of masks contributed to a hidden burden of COVID-19 infection. Furthermore, we hypothesized that restrictive pandemic policies had substantial deleterious impacts on the mental health of people living in the jails.

## Methods

### Overview and Study Design

Between July 8, 2020 and April 30, 2021, we enrolled individuals living and working in four jails in San Mateo County and Santa Clara County, California to participate in a cross-sectional study consisting of SARS-CoV-2 antibody testing and a self-report questionnaire. This study was approved by the Stanford University and Valley Medical Center Institutional Review Boards (protocol #56169 and #20-022, respectively).

### Participant Population and Recruitment

In response to the pandemic, both San Mateo County and Santa Clara County implemented an emergency bail schedule and arrest reductions in order to de-densify their jails, resulting in jail populations of ~520 and 2,000 incarcerated individuals, respectively. The staff population–including custody staff, health care workers, and program staff–remained relatively stable, with approximately 480 and 1,050 staff members in San Mateo County and Santa Clara County, respectively. Incarcerated individuals were recruited through flyers and announcements in their housing units; in single-cell units, recruitment was done door-to-door. Research assistants (RAs) recruited from each housing unit in each jail at least once during the study period, with the exception of isolation units for COVID-19-positive individuals, units deemed by custody staff to be of high security risk, and units housing people with severe mental illnesses. Staff were recruited through flyers posted at work, emails sent by custody health officials, and radio announcements by custody leadership. RAs obtained written informed consent from all participants and emphasized that participation in any part of the study was voluntary with no compensation for participating nor penalty for refusal to participate or for withdrawing from the study. Due to timelines for administrative approvals, enrollment periods in each county were relatively distinct, with enrollment in Santa Clara County beginning only in January 2021 (Supplementary Figure S1). Details on sampling and representativeness are provided in Supplementary Methods and Supplementary Table S1.

### Antibody Testing

To determine the prevalence of prior SARS-CoV-2 infection among residents and staff, we performed antibody testing on finger-prick blood samples using the RightSign COVID-19 IgG/IgM Rapid Test Cassette manufactured by Hangzhou Biotest Biotech, granted emergency use authorization by the FDA (54). All jails had an up-to-date Clinical Laboratory Improvement Amendments (CLIA) certificate of waiver. In an internal validation of test performance using serum samples from a separate study of COVID-19-positive patients (true positives), the RightSign Rapid Antibody Test had 81.5% and 92.1% sensitivity on patient samples from the day of and 28th day following COVID-19 diagnosis, respectively (55). Conversely, it had 100% specificity on 50 serum samples collected prior to 2019 (true negatives). Incarcerated participants received a hard copy of their antibody results on the day of sample collection; staff received results over secure email within 1–2 days. All participants were provided with an informational flier in English or Spanish on how to interpret their antibody test results and the option of consultation with study staff regarding their results.

### Self-Report Questionnaire

We developed separate self-report questionnaires for incarcerated participants and staff participants. The questionnaire for incarcerated participants included the following sections and variables:

1. Demographic and carceral characteristics: age, gender, race/ethnicity, housing, comorbidities including substance misuse, time incarcerated, number of cell mates, COVID-19 vaccination history
2. Infection control policies in practice: reporting of recent flu-like illness, actions taken in response to reporting of illness, COVID-19 test history, frequency of access to new masks (cloth or surgical)
3. Perceptions surrounding COVID-19 and access to care: perceived likelihood of prior infection, fear of getting COVID-19, perceived ability to protect oneself from COVID-19, perceptions toward jail's pandemic response, perceptions of whether health concerns and needs would be recognized and fulfilled in and out of jail
4. Impacts of the COVID-19 pandemic on mental health, routine health care, and court dates

The staff questionnaire included the following sections and variables:

1. Demographic and employment characteristics: age, gender, race/ethnicity, housing, comorbidities, contact with incarcerated individuals at work, health care worker status, COVID-19 vaccination history
2. Perceptions surrounding COVID-19: perceived likelihood of prior infection, fear of getting COVID-19, perceived ability to protect oneself from COVID-19, perceptions toward jail's pandemic response
3. Impacts of the COVID-19 pandemic on mental health

Detailed information on questionnaire variables is provided in Supplementary Methods. For participants whose age, length of time incarcerated, or number of cell mates was unknown, we imputed these variables using all other variables from the questionnaire as detailed in Supplementary Methods.

For incarcerated participants, RAs administered the questionnaire using an electronic tablet. Incarcerated participants could choose to read and respond to questions themselves or to respond orally to questions read aloud. Spanish-speaking RAs and Spanish, Chinese, and Vietnamese text translations were available. To increase privacy, study procedures were conducted in a separate multi-purpose room within each housing unit. Staff participants completed the questionnaire online. Questionnaire data were recorded in a HIPAA-secure REDCap database (56).

### Community Advisory Board and Stakeholder Engagement

Two community advisory boards (CABs), one in each county, guided the overall design and implementation of this study. The goal of the CABs was to ensure that all parts of the study were relevant and sensitive to incarcerated individuals and community stakeholders. In each county, the CAB consisted of people who were currently incarcerated in the jails, representatives from custody health, community organizers from a local advocacy organization, a national advocate for criminal justice reform, and in Santa Clara County, a public defender. Participation in the CAB was voluntary and non-binding. The Stanford study team met with each CAB periodically throughout the study via video conferencing, during which researchers provided an overview of the study aims, design, and procedures and solicited feedback and suggestions from the CAB. Meeting notes were circulated to CAB members following each meeting. In addition to CAB meetings, research staff conducted focus group discussions in various housing units within the jails prior to the enrollment start date as well as halfway through the study.

Of note, CAB and focus group discussions were not transcribed nor analyzed as qualitative data. Rather, they were intended to provide greater transparency into the research process, address questions or concerns regarding the study, and solicit important feedback from stakeholders on five major components of the study: recruitment, enrollment, questionnaire design, results interpretation, and results dissemination. For each of these components, we describe examples of CAB insights and how they were incorporated in the study in Supplementary Table S2. Key insights surrounding results interpretation are also presented as context throughout the Discussion.

### Statistical Analysis

For all seroprevalence analyses, we excluded 82 incarcerated participants and 77 staff participants who were vaccinated prior to enrollment, based on self-reported vaccination status and/or Correctional Health data on vaccine uptake in custody, accessed as previously described (39). We calculated 95% confidence intervals for seroprevalence using the Wilson method for binomial data (57).

For incarcerated participants, we fit multivariate logistic regression models to examine the association between seroprevalence and predictors of interest. Model 1 included only demographic and carceral variables (county, gender, age, race/ethnicity, secure housing before incarceration, length of time incarcerated, and number of cell mates). Model 2 adjusted for demographic and carceral variables and examined perceived likelihood of prior infection as the main predictor of interest. Model 3 adjusted for demographic and carceral variables and examined frequency of access to new masks as the main predictor of interest. Ninety-seven participants who reported previously testing positive for COVID-19 and 189 participants who did not answer the mask access question were excluded from Model 2 and Model 3, respectively. Finally, we fit additional multivariate logistic regression models, adjusted for demographic and carceral variables, to test associations between seroprevalence and perceptions surrounding COVID-19 or barriers to care.

For staff participants, we fit a multivariate logistic regression model for seroprevalence with the following explanatory variables: county, gender, age, race/ethnicity, health care worker status, and contact with incarcerated individuals at work. All analyses were performed in R 4.1.3.

## Results

### Study Population

We enrolled 788 incarcerated individuals and 380 staff members across four jails in adjacent Northern California counties. This sample represented 31% and 25% of the average daily resident and staff population, respectively, across both counties combined. The incarcerated participant population was mostly male (89%), between the ages of 18 and 49 (85%), and Hispanic/Latinx or non-Hispanic Black (66%). Approximately three in ten reported unstable housing prior to incarceration, and 43% reported at least one medical condition considered a potential COVID-19 comorbidity (Table 1). The median and interquartile range (IQR) for length of time incarcerated was 80 days (IQR 15–285). The median number of cell mates was one (IQR 0–7).

**Table 1:** Characteristics of study participants.

	**Incarcerated (*N =* 788)**	**Staff (*N =* 380)**
**County (%)**		
San Mateo County	424 (53.8)	213 (56.1)
Santa Clara County	364 (46.2)	167 (43.9)
**Gender (%)**		
Men	703 (89.2)	199 (52.4)
Transgender/gender non-conforming	6 (0.8)	1 (0.3)
Women	79 (10.0)	180 (47.4)
**Age (%)**		
18–29	252 (32.0)	49 (14.8)
30–49	419 (53.2)	179 (47.1)
50+	114 (14.5)	102 (26.8)
Unknown	3 (0.4)	50 (13.2)
**Race/ethnicity (%)**		
Asian	53 (6.7)	68 (17.9)
Black	147 (18.7)	16 (4.2)
Hispanic/Latinx	376 (47.7)	121 (31.8)
White	83 (10.5)	62 (16.3)
Other/Unknown	129 (16.4)	113 (29.7)
**Comorbidities (%)**		
One or more of:	335 (42.5)	110 (28.9)
Asthma	113 (14.3)	40 (10.5)
Diabetes	51 (6.5)	24 (6.3)
Heart disease or hypertension	61 (7.7)	34 (8.9)
Obesity	40 (5.1)	27 (7.1)
Substance misuse	191 (24.2)	0
Other	44 (5.6)	17 (4.5)
None of the above	383 (48.6)	183 (28.9)
Prefer not to answer	70 (8.9)	87 (22.9)
Already COVID-19 vaccinated (%)	82 (10.4)	77 (20.3)
**Stable housing (%)**		
Yes	521 (66.1)	351 (92.4)
No	235 (29.8)	1 (0.3)
Prefer not to answer	32 (4.1)	28 (7.4)
**Median days incarcerated (IQR)**	80 (15–285)	
**Median number of cellmates (IQR)**	1 (0–7)	
**Health care worker (%)**		
Yes		134 (35.3)
No		216 (56.8)
Prefer not to answer		30 (7.9)
**Contact with incarcerated individuals (%)**		
No		48 (12.6)
Yes		299 (78.7)
Prefer not to answer		33 (8.7)

*Percentages may not sum to 100 due to rounding. Other comorbidities not listed included cancer, immunosuppression, kidney or liver disease, chronic lung disease or COPD. IQR, interquartile range*.

The staff participant population was approximately half women (47%) and mostly 30 years of age and older (74%), with a plurality identifying as Hispanic/Latinx (32%). Approximately three in ten staff participants reported at least one potential COVID-19 comorbidity. Most staff participants (79%) indicated contact with incarcerated individuals at work, and 35% identified as health care workers.

### Prevalence of SARS-CoV-2 Antibodies by Demographic Characteristics

First, we examined the prevalence of antibodies to SARS-CoV-2 (IgG and/or IgM) and its association with demographic or carceral characteristics among people living and working in the jails. In our sample, 13% (88/690; 95% CI, 10–15%) of incarcerated participants (Table 2) and 8% (24/292; 95% CI, 6–12%) of staff participants tested positive for SARS-CoV-2 antibodies (Table 3). After adjusting for other demographic characteristics, antibody positivity was significantly higher in Santa Clara County than San Mateo County, with an adjusted odds ratio (AOR) of 3.3 (95% CI 1.8–6.2) for incarcerated participants and 5.5 (95% CI 2.0–15.0) for staff participants (Tables 2, 3). However, this difference may have been confounded by the later enrollment start in Santa Clara County (Supplementary Figure S1). Among incarcerated participants, other factors associated with higher antibody positivity were Asian race (AOR = 5.7, 95% CI 1.5–21.0) and having eight or more cell mates (AOR = 1.8, 95% CI 1.0–3.3) (Table 2). Among staff, women had significantly lower odds of antibody positivity than men (AOR = 0.3, 95% CI 0.1–0.8) (Table 3).

**Table 2:** Prevalence of antibodies to SARS-CoV-2 among incarcerated participants and association with demographic characteristics, perceived likelihood of prior infection, and access to masks.

	**% Antibody positive (N/Total)**	**Adjusted OR (95% CI)**
**County**		
San Mateo County	5.6 (22/394)	Ref
Santa Clara County	22.3 (66/296)	3.3 (1.8–6.2)***
**Gender**		
Men	12.4 (77/620)	Ref
Transgender/gender non-conforming	0 (0/6)	0 (NA)
Women	17.2 (11/64)	1.4 (0.6–3.0)
**Age**		
18–29	13.6 (31/228)	Ref
30–49	12.8 (47/368)	1.0 (0.6–1.7)
50+	10.9 (10/92)	1.0 (0.4–2.2)
Unknown	0 (0/2)	
**Race/ethnicity**		
Other/Unknown	5.6 (4/72)	Ref
Asian	23.8 (10/42)	5.7 (1.5–21.0)**
Black	7.5 (10/134)	1.6 (0.4–5.5)
Hispanic/Latinx	15.9 (52/328)	3.0 (1.0–9.4)
White	10.5 (12/114)	2.2 (0.7–7.8)
**Secure housing**		
Yes	13.5 (61/451)	Ref
No	9.4 (20/213)	0.7 (0.4–1.2)
Prefer not to answer	26.9 (7/26)	3.0 (1.0–8.7)*
**Length of time incarcerated**		
<30 days	11.6 (26/225)	Ref
30–183 days	12.5 (26/208)	0.7 (0.4–1.4)
184 + days	15.3 (31/203)	0.9 (0.5–1.7)
Unknown	9.3 (5/54)	
**Number of cell mates**		
0–1	9.8 (40/410)	Ref
2–07	7.2 (9/125)	0.9 (0.4–2.0)
8+	25.3 (38/150)	1.8 (1.0–3.3)*
Unknown	20.0 (1/5)	
**Perceived likelihood of prior infection**		
Very unlikely/unlikely	3.9 (14/355)	Ref
Possible	5.1 (8/158)	1.3 (0.5–3.2)
Likely/very likely	24.5 (13/53)	8.9 (3.6–22.0)***
Prefer not to answer	14.8 (4/27)	1.6 (0.4–6.7)
I tested positive for COVID-19	50.5 (49/97)	
**Access to new masks**		
Once a week	1.9 (1/54)	Ref
Less than once a week	18.4 (77/419)	13.8 (1.8–107.0)*
Prefer not to answer	17.9 (5/28)	9.2 (1.0–89.4)
**Flu-like illness since Feb 2020**		
Sick in jail, reported symptoms	42.4 (28/66)	
Sick in jail, did not report symptoms	16.7 (7/42)	
Sick outside of jail	20.3 (16/79)	
None	6.6 (32/483)	
Prefer not to answer	25.0 (5/20)	
**Total**	12.8 (88/690)	

*Participants already vaccinated at the time of enrollment were excluded. Adjusted odds ratios (ORs) from Model 1 are shown for demographic and carceral characteristics (county, gender, age, race/ethnicity, secure housing, length of time incarcerated, number of cell mates). Adjusted ORs from Model 2 and Model 3 are shown for perceived likelihood of past infection and access to new masks, respectively. CI, confidence interval.*

*p <0.05.

**p <0.01.

***p <0.001.

**Table 3:** Prevalence of antibodies to SARS-CoV-2 among staff participants and association with demographic and employment characteristics.

	**% Antibody positive (N/Total)**	**Adjusted OR** **(95% CI)**
**County**		
San Mateo County	3.4 (7/207)	Ref
Santa Clara County	20.0 (17/85)	5.5 (2.0–15.0)***
**Gender**		
Men	10.9 (18/165)	Ref
Transgender/gender non-conforming	0 (0/1)	0 (NA)
Women	4.8 (6/126)	0.3 (0.1–0.8)*
**Age**		
18–29	10.0 (4/40)	Ref
30–49	10.0 (14/140)	0.6 (0.2–2.2)
50+	1.4 (1/70)	0.1 (0–1.2)
Unknown	11.9 (5/42)	
**Race/ethnicity**		
Other/unknown	14.8 (8/54)	Ref
Asian	2.6 (1/38)	0.3 (0–2.8)
Black	18.2 (2/11)	1.7 (0.2–14.1)
Hispanic/Latinx	8.5 (8/94)	0.8 (0.2, 3.6)
White	5.3 (5/95)	0.5 (0.1–2.5)
**Health care worker**		
Yes	4.8 (4/83)	Ref
No	8.2 (15/183)	0.9 (0.2, 3.4)
Prefer not to answer	19.2 (5/26)	5.3 (0–1167.1)
**Contact with incarcerated individuals**		
No	4.9 (2/41)	Ref
Yes	7.7 (17/222)	0.9 (0.2–4.6)
Prefer not to answer	17.2 (5/29)	0.1 (0–40.6)
**Flu-like illness since Feb 2020**		
Yes	8.8 (5/57)	
No	6.8 (14/205)	
Prefer not to answer	16.7 (5/30)	
**Perceived likelihood of past infection**		
Very unlikely/unlikely	2.0 (2/102)	
Possible	4.6 (5/108)	
Likely/very likely	10.0 (4/40)	
Prefer not to answer	17.2 (5/29)	
I tested positive for COVID-19	61.5 (8/13)	
**Total**	8.2 (24/292)	

*Participants already vaccinated at the time of enrollment were excluded. OR, odds ratio; CI, confidence interval.*

*p <0.05.

***p <0.001.

### Contributors to Undetected COVID-19 Infection

To assess the extent of undetected COVID-19 infection in the jails, we compared participants' antibody test results with self-reported history of a positive COVID-19 test. Nearly half (39/88; 44%) of incarcerated participants who were antibody-positive did *not* report a prior COVID-19 diagnosis (Table 2). Among these antibody-positive incarcerated participants without a prior COVID-19 diagnosis, 46% reported having flu-like illness since February 2020 (31% outside jail, 15% in jail). To test our hypothesis that the hidden burden of infection was attributable in part to inadequate responses to reported illness or symptom underreporting, we analyzed responses from 123 (16%) incarcerated participants who reported having flu-like illness in jail since February 2020 (Table 2). Among participants who reported their symptoms to jail staff, only 62% indicated getting tested for COVID-19 and over one in five (22%) indicated that no action was taken (Table 4). Moreover, 39% of participants who were sick in jail did not report their symptoms to jail staff. The leading reason for symptom underreporting was not thinking it was not serious enough to report (47%), followed by not thinking anything would be done about it (28%), concern about being put in isolation (26%), and worry about how jail staff would treat them (21%) (Table 4).

**Table 4:** Reporting of illness and access to face masks among incarcerated participants.

	**% Incarc respondents**
**Did you report your symptoms to jail staff? (Among participants who had flu-like illness in jail)**	
Yes	60.7
No	39.3
**What action was taken when you reported your symptoms? (Among participants who responded “Yes” to the first question) (Select all that apply)**	
I was tested for COVID-19	62.1
I was put in isolation	51.7
I was taken to the medical clinic for evaluation	34.5
No action was taken	22.4
**Why didn**'**t you report your symptoms? (Among participants who responded “No” to the first question) (Select all that apply)**	
I didn't think it was serious enough to report	46.8
I didn't think anything would be done about it	27.7
I was concerned about being put in isolation	25.5
I was worried about how staff in the jail would treat me	21.3
I was worried about how other incarcerated people would treat me	2.1
Other	12.8
**How often do you get a new mask? (Among participants incarcerated for at least 30 days)**	
Once a week	7.3
Once a month	17.5
Less frequent than once a month	40.2
I have only received **one** mask since the start of the pandemic	33.9
I do not have one	1.0

*Percentages were calculated after excluding those with missing or “prefer not to answer” responses and may not sum up to 100 due to rounding*.

We next utilized multivariate logistic regression to examine the association between antibody positivity and perceived likelihood of prior infection among individuals without a prior COVID-19 diagnosis. We reasoned that a positive association would indicate that antibody-positive individuals who were aware of COVID-19 exposure or infection did not get tested, providing further evidence that limited access to testing and deterrents to symptom reporting or testing uptake contributed to undetected infection. After adjusting for demographic and carceral characteristics, the odds of prior infection were 8.9 (95% CI, 3.6–22.0) times higher among participants who thought it was likely or very likely that they had COVID-19, compared to participants who thought it unlikely or very unlikely (Table 2).

Of note, we also found undetected infection among staff, with only one-third of antibody-positive staff participants reporting a previous positive COVID-19 test (Table 3). Among the remaining two-thirds of antibody-positive staff who did not report a prior COVID-19 diagnosis, 13% reported having flu-like illness since February 2020. We were underpowered to test the association between antibody positivity and perceived likelihood of prior infection among staff participants.

### Limited Access to Masks and Association With Infection Risk

Throughout the pandemic, face masks have been one of few ways in which incarcerated individuals have been able to protect themselves from COVID-19. Indeed, when incarcerated participants were asked to select three things that would protect them most from COVID-19, face masks were a leading protective measure cited by 56% of participants, second only to release from jail (75%) (Supplementary Table S3). However, we found that access to new masks for jail residents was extremely limited: among participants incarcerated for at least 30 days, only 7% received a new mask once a week and 17% once a month (Table 4). Alarmingly, nearly three-quarters of participants reported receiving a new mask less often than once a month.

To test our hypothesis that limited mask access was associated with increased risk of prior infection, we again used multivariate logistic regression to assess the association between antibody positivity and mask access, adjusting for demographic and carceral factors. Restricted access to masks—defined as receiving a new mask less often than once a week—was associated with significantly higher odds of prior infection (AOR 13.8, 95% CI 1.8–107.0) (Table 2).

### Perceptions Surrounding COVID-19 and Barriers to Care

Among incarcerated participants, we identified prevalent experiences of frequent stress or fear around getting COVID-19 in jail (39% of participants), perceptions of being unable to protect oneself from COVID-19 in jail (54% of participants), and perceptions that not enough was being done to protect incarcerated individuals from COVID-19 (58% of participants) (Supplementary Table S3). We also identified pervasive perceptions of barriers to health care in jail, with only 23% and 35% of incarcerated participants who believed that their health concerns were taken seriously by correctional officers or jail health staff, respectively (Supplementary Table S4). This mistrust appeared setting-specific, as 60% of incarcerated participants believed that their health concerns were taken seriously by their doctor outside of jail. Similarly, 43% of incarcerated participants expressed concerns of being denied medical treatment or services while incarcerated, compared to 27% who expressed concerns of being denied treatment outside of jail (Supplementary Table S4). We tested whether any of these perceptions were associated with antibody prevalence. After adjusting for demographic and carceral characteristics, neutrality or disagreement regarding whether one's health concerns were taken seriously by jail health staff was associated with 2.1 (95% CI 1.0–4.5) increased odds of seropositivity, compared to those who agreed with this statement (Supplementary Table S5).

In contrast, an overwhelming majority of staff participants (95%) felt at least somewhat able to protect themselves from COVID-19 while at work (Supplementary Table S3). While only 20% reported experiencing frequent stress or fear around getting COVID-19 at work, 39% did report frequent stress or fear around bringing COVID-19 from work to others in their household or community. When asked whether enough was being done to protect incarcerated individuals from COVID-19, 67% of staff participants agreed or strongly agreed (Supplementary Table S3). When asked whether enough was being done to protect *staff* from COVID-19, 51% of staff participants agreed or strongly agreed.

### Impacts of COVID-19 on Court Dates, Mental Health, and Routine Health Care

Among incarcerated participants, 61% indicated that their court dates were impacted by the COVID-19 pandemic. Delays (76%), limits on attendance (56%), and cancellations (39%) were the most common impacts cited (Supplementary Table S6). Notably, among participants whose court dates were delayed, 44% reported delays of over 2 months (Supplementary Table S6).

The COVID-19 pandemic also had impacts on mental health, with 38% of incarcerated participants citing worse mental health due to the pandemic (Table 5). Leading reasons for worsened mental health were lack of connection to family and other loved ones (75%) and fear of getting COVID-19 (67%) (Table 5). Other common reasons included limits on programming (ie., classes, support groups) (56%), changes in recreation time (56%), unsanitary/unsafe conditions (56%), family or personal issues (55%), financial insecurity due to COVID-19 (46%), and lack of information about COVID-19 (39%). Our findings also revealed impacts on routine mental or physical health care in jail. Of the 38% and 43% incarcerated participants who reported previously receiving regular mental or physical health care in jail, respectively, approximately 40% said their health care had decreased or stopped due to the pandemic (Supplementary Table S6).

**Table 5:** Impacts of the COVID-19 pandemic on mental health and reasons for worsened mental health among incarcerated and staff participants.

	**% Incarc respondents**	**% Staff respondents**
**How has your mental health been impacted by COVID19?**		
It has been better	1.9	1.7
It has been better	4.0	5.7
My mental health has not been affected	45.0	60.1
It has been worse	23.5	22.8
It has been much worse	14.8	3.4
Prefer not to answer	10.9	6.3
**What do you think has affected your mental health while in custody during COVID-19? (Among incarcerated participants with worsened mental health) (Select all that apply)**		
Lack of connection to family and other loved ones	75.4	
Fear of getting COVID-19	66.5	
Lack of programs due to COVID-19 (i.e. classes, support groups)	56.4	
Changes in recreation time due to COVID-19	55.9	
Unsanitary/unsafe conditions	55.9	
Family or personal issues	55.1	
Financial insecurity due to COVID-19	45.8	
Lack of information about COVID-19	39.4	
Other	12.7	
Prefer not to answer	1.3	
**What do you think has affected your mental health while in working in a correctional facility during COVID-19? (Among staff participants with worsened mental health) (Select all that apply)**		
Fear of getting COVID-19		63.8
Unsanitary/unsafe conditions		44.7
Family or personal issues		42.6
Lack of information about COVID-19		25.5
Frequency of COVID-19 routine testing		23.4
Other		8.5
Nothing		6.4
Prefer not to answer		4.3

*Percentages were calculated after excluding those with missing responses and may not sum up to 100 due to rounding*.

Among staff participants, over one quarter reported worsened mental health due to the COVID-19 pandemic, with leading reasons including fear of getting COVID-19 (64%), unsanitary/unsafe conditions at work (45%), family or personal issues (43%), lack of information about COVID-19 (26%), and frequency of COVID-19 routine testing (23%) (Table 5).

## Discussion

In this study across four Northern California county jails, antibody testing revealed a hidden COVID-19 burden among people living and working in the jails. By pairing antibody data with questionnaire responses, we found that undetected infection was concentrated among jail residents who suspected prior infection but remained undiagnosed, which may have been due in part to symptom underreporting and/or inaction by staff in response to reported illness. Residents also indicated deficient access to face masks, which was strongly associated with increased risk of prior infection. Perceptions of medical neglect in jail were prevalent among residents, as well as experiences of worsened mental health due to restrictive COVID-19 policies. Together, these findings shed light on practical barriers to infection prevention and control in carceral settings and underscore the need for improved implementation of preventive measures as well as a pandemic response strategy that minimizes harm to mental health and well-being.

To our knowledge, this study was the first to employ antibody testing in a U.S. carceral setting. Among residents, dormitory-style housing was associated with increased risk of prior infection, corroborating prior work in prisons (58). In concordance with previous accounts of under-testing in prisons and jails (25–28), this study revealed substantial undetected COVID-19 infection among both residents and staff. These results are consistent with other studies employing antibody testing in carceral (31) and non-carceral settings (30, 59). We also found a significant association between antibody positivity and perceived likelihood of prior infection among residents without a prior COVID-19 diagnosis, suggesting that the hidden burden of infection was concentrated among individuals who were aware of exposure or infection but had not been tested. There could be several reasons for this, including limited access to testing and/or deterrents to testing uptake. While we were underpowered to directly assess whether and to what extent these two factors contributed to undetected infection, we did find evidence for the standalone existence of both phenomena.

First, regarding limited access to testing, we found that even among residents who reported their flu-like illness to jail staff, only 62% said they were then tested for COVID-19, and 22% said no action was taken. Relatedly, many residents believed that their health concerns were neglected by jail staff; this belief may reflect institutional or medical mistrust that could impede care-seeking or uptake of other preventive measures like vaccination, as has been shown in other studies (39, 46, 60). In particular, residents who were neutral or in disagreement about jail health staff taking their health concerns seriously had increased odds of antibody positivity; however, we were unable to infer causality or to determine the direction of causation. Regardless, these collective findings illustrate the need for more systematic, consistent, and transparent protocols for responding to residents' reported illness and other health concerns.

Second, regarding deterrents to testing uptake, we found that nearly four in ten individuals who had flu-like illness in jail did not report their symptoms to jail staff. Reasons cited for symptom underreporting included beliefs that nothing would be done about it and fears of being placed in isolation. Accordingly, incarcerated members of our CAB cited widespread fears that a positive COVID-19 test would effectively lead to solitary confinement. Considered in conjunction with evidence on the health harms of restrictive housing (11–13), these findings strongly caution against over-reliance on isolation and quarantine in place of comprehensive implementation of other preventive measures such as masking, testing, and vaccination for residents and staff. When medical isolation is necessary, jail administrators and staff should undertake exhaustive efforts to distinguish its conditions from solitary confinement, which could critically reduce barriers to reporting of illness. This could include providing individuals in isolation with free and enhanced access to entertainment, nutritious meals, outdoor time, phone and video calls with loved ones, and frequent oversight and status updates from healthcare staff (43, 47).

Incarcerated participants also indicated extremely limited access to new masks, which we found to be significantly associated with elevated infection risk as measured by antibody positivity. Of note, although the importance of proper mask wearing is well-understood, our incarcerated stakeholder representatives drew attention to the overlooked issue of mask maintenance and replacement. Namely, they reported peers having torn masks from overuse and spoke of being unable to wash their soiled cloth mask without another to wear while it dried. While the jails' official policy was to provide new masks for residents upon request, our findings highlight the need for an active rather than passive approach to periodic mask distribution and/or laundering, and generally for more systematic, consistent, and transparent protocols for responding to residents' reported illness and other health concerns.

This study also revealed detrimental impacts of the pandemic on residents' cases and mental health. Our finding of pervasive court delays and cancellations substantiates a recent investigation which uncovered severe case backlog in California that has only been exacerbated by the pandemic (61). Many residents also cited restrictions on court attendance; as our CAB pointed out, these restrictions hindered participatory defense, a community organizing model developed locally that engages family and community members in shaping a loved one's case (62). In addition to case-related stressors, we identified prevalent worsened mental health among residents that was attributed not only to fear of COVID-19 and unsanitary conditions but also to restrictive pandemic policies, corroborating prior qualitative work (45, 63–65). These mental health harms have likely only intensified with prolonged restrictions: all four jails suspended in-person visitation for over 10 months, and some continue to restrict recreation time and in-person programming over 2 years into the pandemic. While these measures can help mitigate transmission during major outbreaks, their prolonged and unnecessary use violates minimum human rights standards (3, 66, 67) and, as our findings warn, may be contributing to a second crisis of mental health among residents. Therefore, administrators should ensure prompt resumption and continuation of in-person visitation, programs, and standard recreation time, especially when facility and community transmission is low (68).

While this study focused largely on incarcerated individuals, we also identified various topics of interest relating to jail staff that merit future study. Male staff had significantly higher odds of antibody positivity even after adjusting for employment type; however, we were underpowered to identify other variables robustly associated with prior infection or undetected infection among staff. Additional research is needed on this topic given its implications for disease spread within carceral facilities and between carceral facilities and outside communities. In addition, the role of jail staff in contributing to or mitigating the deficiencies in infection prevention and control remains unclear. While staff generally felt able to protect themselves from COVID-19 at work, some still reported unsanitary conditions, worsened mental health due to the pandemic, and frequent fears of getting infected at work and bringing it home. These issues may contribute to critical staffing shortages occurring in prisons and jails across the U.S., which have had dire consequences for residents and staff alike (69). However, the pandemic has only further exposed and exacerbated the various threats to public health and human rights long posed by incarceration (1, 2, 14, 67, 70, 71); accordingly, efforts should focus on minimizing the population exposed to carceral settings rather than re-expanding the carceral workforce (15, 69).

This study had several limitations. All questionnaire data were subject to self-report biases; however, for incarcerated participants we validated demographic information and COVID-19 test history with custody records or the jail electronic health record (EHR) when available (Supplementary Methods). We also mitigated social desirability bias by administering questionnaires online for staff or via electronic tablet for incarcerated individuals when possible. Our participant population was likely a biased sample due to voluntary participation, language barriers for non-English or Spanish speakers, and exclusion of people in COVID-19 isolation, people in high security units, and people with severe mental illnesses; moreover, we were unable to track response rate. Therefore, our findings may not be representative of the entire resident or staff population and may have more qualitative value than quantitative precision. Due to small sample size, we did not analyze smaller racial/ethnic subgroups, such as Indigenous/Pacific Islander individuals or Hispanic/Latinx individuals of different races, but future studies should assess differences in infection risk or COVID-19-related perceptions across racial/ethnic subgroups. For logistic regression analyses we imputed missing data on age, length of incarceration, and number of cell mates; using imputed data led to trivial differences compared to excluding observations with missing data. Furthermore, our estimates of the extent of prior and undetected infection are affected by counteracting factors of imperfect test specificity vs. insufficient test sensitivity, lack of seroconversion, and antibody waning. However, these factors likely had similar effects on all strata that we compared. Finally, our findings may have limited generalizability to other carceral facilities but nonetheless reflect challenges that are shared across many carceral settings.

## Conclusions

This study reveals significant practical barriers to achieving infection control in carceral settings. Reported deficiencies in preventive measures and the harmful conditions of medical isolation may foster mistrust and fears that in turn undermine symptom reporting, testing uptake, and vaccine acceptance. Concurrently, restrictive pandemic policies have resulted in heightened social isolation, deprivation, and case-related stress that exacerbate poor mental health and the already distressing experience of incarceration. In the short term, our findings warrant diligent efforts from custody and health officials to transform the conditions of medical isolation and to ensure periodic active mask provision and consistent, transparent responses to residents' reported illness. Custody officials should also prioritize prompt restoration of in-person visitation, programs, and services essential for the health and well-being of people living in carceral facilities. Ultimately, our findings highlight numerous obstacles to maintaining health and human rights in carceral settings and underscore the need for community-based investments to enable sustained decarceration during and beyond pandemic times (72).

## Data Availability Statement

The datasets presented in this article are not readily available because of ethical and privacy concerns but may be made available in de-identified or aggregate form in the future, as allowed by the IRB. Requests to access the datasets should be directed to YL, yiranliu@stanford.edu.

## Ethics Statement

The studies involving human participants were reviewed and approved by the Stanford Institutional Review Board (#56169) and Santa Clara Valley Medical Center Institutional Review Board (#20-022). The patients/participants provided their written informed consent to participate in this study.

## Author Contributions

YL, CL, and JA contributed to conceptualization and study design. MRod, BS, and CT acquired the data. YL wrote the first draft of the manuscript and analyzed the data with critical input from CL and JA. All authors contributed to data interpretation, manuscript revision, and approved the submitted version.

## Funding

This work was supported by the COVID-19 Emergency Response Fund from Stanford University, which was established with a gift from the Horowitz Family Foundation, and by a Clinical and Translational Science Award (#5UL1TR003142) from the National Institutes of Health (NIH) National Center for Advancing Translational Sciences (NCATS). The funders were not involved in any aspect of the study.

## Conflict of Interest

The authors declare that the research was conducted in the absence of any commercial or financial relationships that could be construed as a potential conflict of interest.

## Publisher's Note

All claims expressed in this article are solely those of the authors and do not necessarily represent those of their affiliated organizations, or those of the publisher, the editors and the reviewers. Any product that may be evaluated in this article, or claim that may be made by its manufacturer, is not guaranteed or endorsed by the publisher.
