# Corrigendum: COVID-19 preventive measures in Northern California jails: Perceived deficiencies, barriers, and unintended harms

**DOI:** 10.3389/fpubh.2022.1002199

**Published:** 2022-08-16

**Authors:** Yiran E. Liu, Christopher LeBoa, Marcela Rodriguez, Beruk Sherif, Chrisele Trinidad, Michael del Rosario, Sophie Allen, Christine Clifford, Jennifer Redding, Wei-ting Chen, Lisa G. Rosas, Carlos Morales, Alexander Chyorny, Jason R. Andrews

**Affiliations:** ^1^Division of Infectious Diseases and Geographic Medicine, Department of Medicine, Stanford, CA, United States; ^2^Cancer Biology Graduate Program, Stanford University School of Medicine, Stanford, CA, United States; ^3^Stanford Center for Clinical Research, Stanford University School of Medicine, Stanford, CA, United States; ^4^Division of Correctional Health Services, San Mateo County Health, Redwood City, CA, United States; ^5^Stanford Law School, Stanford, CA, United States; ^6^Department of Sociology, Stanford School of Humanities and Sciences, Stanford, CA, United States; ^7^Silicon Valley De-Bug, San Jose, CA, United States; ^8^Santa Clara County Office of the Public Defender, San Jose, CA, United States; ^9^Office of Community Engagement, Stanford University School of Medicine, Stanford, CA, United States; ^10^Division of Custody Health, Department of Medicine, Santa Clara Valley Health and Hospital System, San Jose, CA, United States

**Keywords:** COVID-19, incarceration, jails, infection control, stakeholder engagement, community-based research, seroprevalence, mental health

In the published article, the **Acknowledgments** section was mistakenly not included in the publication. The **Acknowledgments** section appears below:

## Acknowledgments

We thank all the members of our community advisory board (CAB) and focus groups, including Sarait Escorza, Zachary Kirk, Brendan Harris, Maurice Friera, Erwin Inguillo, Adrian Maldonado, Alexa Ramirez, David Martell, Marco Ruiz, Venitia Hyatt, Victrine Perales, Daniel Lanzarin, Clara Boyden, and Mary Fullerton. We thank Shaka Senghor for his participation in the CAB and advice as a consultant for the study. We thank Taia Wang, Julie Parsonnet, and Kristen Aiemjoy for sharing expertise and resources for serology testing and study materials. We thank Robert Spencer for guidance on study design. We thank Sumana Shashidhar for guidance with the IRB. We thank Hector Romero, Erica Martinez, and Olivia Tigre for support with CAB meetings. We thank Andrea Wang for graphic design of study flyers and handouts. We thank Mark Padget, Theodore Shelton, James Kirkland, Antonio Fernandes, Mark Myers, Roman Mosqueda, John Boy Palarca, John Kovach, and William Fogarty for facilitating access to the jails and facilitating participation of incarcerated individuals in the CAB. We thank Melissa Wagner, Nicole Hayes, Natalie Saavedra, and Iryna Kalish for providing access to data.

The authors apologize for this error and state that this does not change the scientific conclusions of the article in any way. The original article has been updated.

## Publisher's note

All claims expressed in this article are solely those of the authors and do not necessarily represent those of their affiliated organizations, or those of the publisher, the editors and the reviewers. Any product that may be evaluated in this article, or claim that may be made by its manufacturer, is not guaranteed or endorsed by the publisher.

