# Supplementary material for: COVID-19 Preventive Measures in Northern California Jails: Perceived Deficiencies, Barriers, and Unintended Harms
Source: Front Public Health. 2022 Jun 14;10:854343. doi: 10.3389/fpubh.2022.854343 (PMC9237366; doi:10.3389/fpubh.2022.854343)
Supplement: Supplementary file 1 [file Data_Sheet_1.PDF]

I. Supplementary Methods

Sampling method and representativeness
For incarcerated individuals, we used convenience sampling with informal stratification by county, jail, gender, housing unit, and intake status (ie. new admissions versus main population). Research assistants (RAs) visited a different housing unit each day to enroll individuals interested in participating. For staff, we used voluntary response sampling based on email announcements of when RAs would be on-site with a link to an electronic platform for making an appointment to participate in the study. Consequently, we could not track response rates for incarcerated individuals or staff as we were unable to distinguish between non-response due to time constraints of our RAs versus non-response due to lack of willingness to participate. Sample representativeness of incarcerated participants by demographic characteristics is shown in **Table S1**; we were unable to assess sample representativeness for staff participants due to lack of data on the staff population.

Variables. Details on variables included in the main tables and logistic regression analyses are provided below.

Age. We used date of birth to compute participants' age at the time of participation in the study, and grouped age into the following categories: 18-29, 30-49, and 50+.

Race/ethnicity. We asked about race and ethnicity separately in our questionnaire but combined these variables in our analyses based on U.S. Census Bureau recommendations [1]. We categorized race/ethnicity as follows. Participants whose race/ethnicity was missing Individuals with more than one race/ethnicity identification were analyzed as a part of one group only, with Hispanic/Latinx superseding Black, then Indigenous or Pacific Islander, then Asian, then White, then Other/Unknown. Indigenous and Pacific Islander individuals were combined with the Other/Unknown category due to small sample size. We did not explicitly distinguish individuals with two or more racial/ethnic identifications due to lack of consensus guidance around whether Hispanic/Latinx individuals should be considered as having two or more identifications, and due to small sample size.

Stable housing. We asked whether participants were living in stable housing prior to being incarcerated, defined as having consistent access to a place to live.

Length of time incarcerated. We computed days incarcerated at the time of participation in the study based on date of arrival at the jail. We grouped responses into the following categories: <30 days, 30-183 days, and 184+ days.

Number of cell mates. We asked incarcerated participants to identify how many people they sleep in the same room with, not including themselves.

Health care worker. We asked staff participants whether they are a health care worker.

Contact with incarcerated individuals. We asked staff participants whether they have any contact with incarcerated individuals on a typical workday.

Perceived risk of prior infection. We asked participants how likely they thought it was that they already had COVID-19, with options of very unlikely, unlikely, possible, likely, very likely, or indicating that they had tested positive for COVID-19. We categorized responses into very unlikely/unlikely, possible, likely/very likely, and previously testing positive for COVID-19. To better identify participants who had previously tested positive for COVID-19, we integrated responses to this question with responses to a separate question asking whether participants had ever tested positive for COVID-19 by nasal/oral swab test. For incarcerated participants in Santa Clara County, we validated their self-reported COVID-19 test

history with data from the jail electronic health record (EHR). 97 incarcerated participants who previously tested positive for COVID-19 in either question were excluded from Model 3.

Flu-like illness since February 2020. We asked participants whether they had at least one illness with fever, cough, or shortness of breath since February 2020. For incarcerated participants, we asked whether they had this illness while in jail, and whether they reported their symptoms to jail staff.

Access to new masks. Most masks distributed by the jails were cloth masks. We asked incarcerated participants how often they get a new mask, with options of once a week, once a month, less frequent than once a month, having only received one mask since the start of the pandemic, or not having one. For logistic regression analysis, we combined the last four responses into the category "less than once a week."

Missing data. For incarcerated participants whose demographic information (age, gender, race/ethnicity) or incarceration start date were missing, we accessed these variables from their custody record (San Mateo County) or EHR (Santa Clara County) when available. Participants whose race/ethnicity remained missing were categorized as "Other/Unknown." For participants whose age, incarceration start date, and number of cell mates remained missing, we utilized a random forest algorithm (missForest [2]) to impute age for two (0.3%) incarcerated participants and 42 (14.4%) staff participants, length of time incarcerated for 54 (7.8%) incarcerated participants, and number of cell mates for five (0.7%) incarcerated participants, using all other variables from the questionnaire, including demographic and carceral characteristics, history of flu-like illness, perceptions surrounding COVID-19, perceptions towards correctional and medical personnel, unintended impacts of the pandemic.

References

1. Compton, E.B., Michael; Ennis, Sharon; Rastogi, Sonya, *2010 Census Race and Hispanic Origin Alternative Questionnaire Experiment*. 2013, U.S. Census Bureau.
2. Stekhoven, D.J. and P. Bühlmann *MissForest - nonparametric missing value imputation for mixed-type data*. 2011. arXiv:1105.0828.
3. *San Mateo County Jail and Prison Statistics*. 2/8/22]; Data Source: U.S. Bureau of Justice Statistics; National Prisoner Statistics (NPS) Program, Prisoners In 2015.]. Available from: https://www.countyoffice.org/ca-san-mateo-county-jails-prisons/.

II. Supplementary Figures and Tables

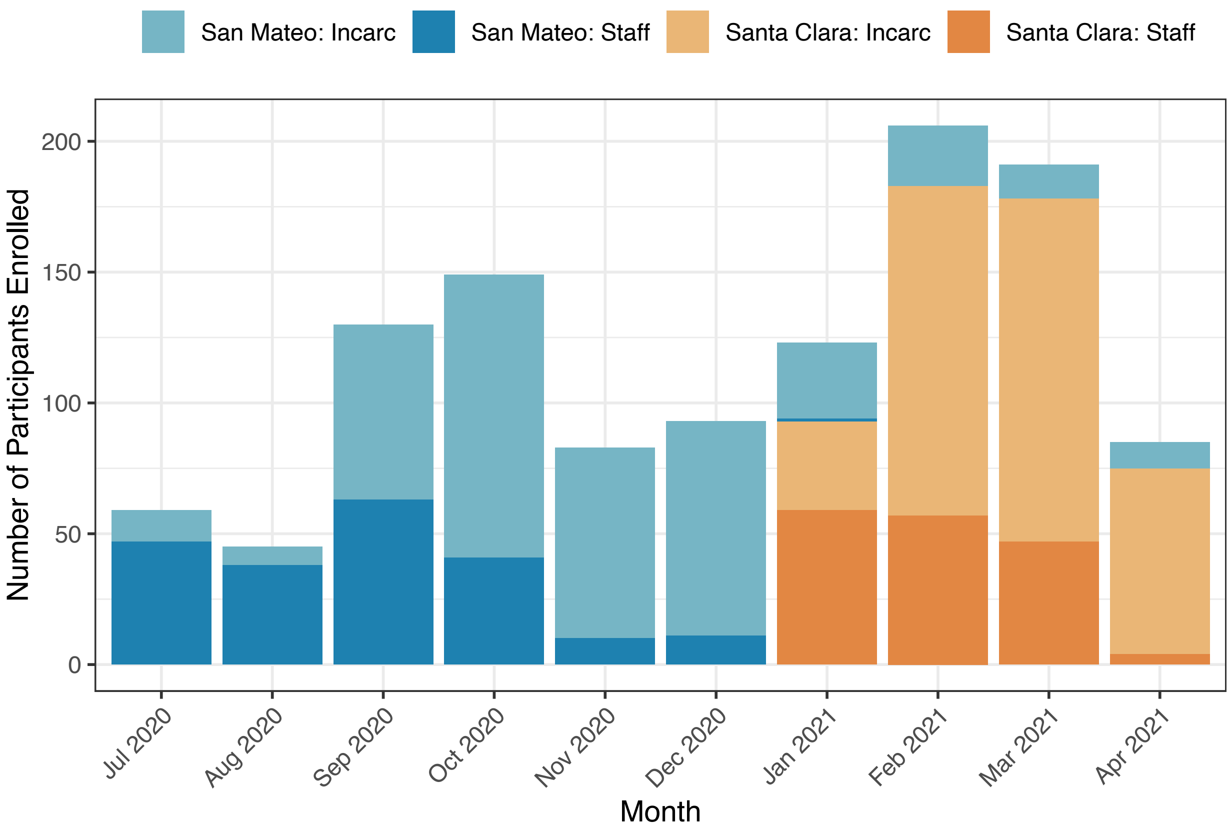

Figure S1. Participants enrolled each month, by county and population. Incarc, incarcerated.

Table S1. Demographics of incarcerated participants compared to the county jail population. All numbers are percentages. Data on Santa Clara County were from April 2021. Data on San Mateo County were from 2015 [3] and did not include age. Percentages may not sum up to 100 due to missing data or rounding.

	Incarcerated Participants (%)	Santa Clara County Jail Population (%)	San Mateo County Jail Population (%)
**Gender**			
Men	89.2	90.5	86.9
Transgender / gender non-conforming	0.8		
Women	10.0	9.5	11.1
**Age**			
18-29	32.0	28.6	
30-49	53.2	56.3	
50+	14.5	15.1	
**Race/Ethnicity**			
Asian	6.7	7.6	7.0
Black	18.7	11.7	22.6
Hispanic/Latinx	47.7	45.6	36.7
White	10.5	23.0	25.9
Other/Unknown	16.4	12.1	7.7

Table S2. Study components informed by the Community Advisory Board (CAB). Examples of insights provided by the CAB and corresponding actions taken by the study team are shown for each component of the study.

**Study Component**	**Example CAB Insight**	**Example Response from Study Team**
Recruitment	Repeated presence of study team in housing units will help foster trust and increase participation; hearing from peers (especially pod workers or leaders) about experience in study will help overcome hesitations about participating	Research assistants re-visited housing units over several weeks or months to have discussions with and recruit individuals who became more interested in participating in the study over time
Enrollment	Concerns about antibody test results affecting housing or participation in programming	Research assistants emphasized during informed consent process that antibody test results would be confidential and would not be used for any decision making by the jail
Questionnaire design	Reports of varied access to masks and lack of response to illness	Study team added CAB-recommended questions about frequency of access to new masks and jail staff's response to symptom reporting
Results interpretation	Fears of testing positive for COVID-19 and experiencing extreme conditions of medical isolation, causing lockdown and suspension of programming for entire pod	Study team included this in manuscript and presentations as context for results showing a hidden burden of infection and underreporting of symptoms
Results dissemination	Findings should be shared with local policymakers, news outlets, and incarcerated individuals and their loved ones	Study team has shared and will continue sharing findings with county supervisors, local news outlets, and local community organizations and stakeholders through email communications, policy briefs or brochures, and presentations

Table S3. Perceptions surrounding protection from COVID-19 among incarcerated and staff participants. Percentages were calculated after excluding those with missing responses and may not sum up to 100 due to rounding.

	% Incarc respondents	% Staff respondents
**How well do you feel you can protect yourself from COVID-19 while in jail / at work?**		
Able or very well able	16.8	51.5
Somewhat able	29.2	43.8
Not really or not at all able	54.0	4.8
**What are three things that you feel help protect you most from COVID-19?**		
Release from jail	75.2	
Face masks/PPE	55.7	
Sanitizing shared spaces	42.9	
Washing hands with soap	37.9	
Physical distancing	34.5	
Staff wearing masks/PPE	28.9	
Access to testing	28.3	
Better medical care	24.8	
More contact with loved ones outside	12.7	
Commissary	7.1	
Other	4.4	
Prefer not to answer	3.5	
**How often have you experienced stress, fear, worry, and/or anxiety about getting COVID-19 in jail / at work?**		
Often or all the time	38.9	20.1
Sometimes	23.5	27.4
Occasionally or never	37.7	52.5
**How often have you experienced stress, fear, worry, and/or anxiety about bringing COVID-19 infection from work to others in your household or in your community?**		
Often or all the time		39.3
Sometimes		25.8
Occasionally or never		34.9
**Enough is being done to protect incarcerated individuals from COVID-19**		
Strongly agree	5.5	22.5
Agree	15.0	44.4
Neutral	21.3	21.9
Disagree	23.9	7.5
Strongly disagree	34.3	3.6
**Enough is being done to protect jail staff from COVID-19**		
Strongly agree		14.9
Agree		36.3
Neutral		30.4
Disagree		13.4
Strongly disagree		5.1

Table S4. Concerns about barriers to health care in and out of custody among incarcerated participants. Percentages may not sum up to 100 due to rounding.

	**I believe that ______ take(s) my health concerns seriously.**			**While _____, I worry that I will be denied the treatment or services I need.**	
	**The correctional officers**	**The jail doctors and nurses**	**My doctor outside of jail**	**In jail/prison**	**Outside of jail/prison**
**Strongly Agree**	7%	10%	20%	18%	8%
**Agree**	16%	25%	40%	25%	19%
**Neutral**	27%	30%	24%	28%	23%
**Disagree**	20%	16%	6%	16%	30%
**Strongly Disagree**	23%	14%	6%	6%	13%
**Prefer not to answer**	6%	6%	5%	6%	7%

Table S5. Results of additional multivariate logistic regression models examining the association of seropositivity with perceptions surrounding COVID-19 or barriers to care. All models were adjusted for demographic and carceral characteristics. OR, odds ratio; *p<0.05.

	**Adjusted OR (95% CI)**
**How well do you feel you can protect yourself from COVID-19 while in jail / at work?**	
Able or very well able	Ref
Somewhat able	1.2 (0.5-3.0)
Not really or not at all able	1.6 (0.7-3.7)
**How often have you experienced stress, fear, worry, and/or anxiety about getting COVID-19 in jail / at work?**	
Occasionally or never	Ref
Sometimes	1.4 (0.7-2.7)
Often or all the time	1.0 (0.5-1.8)
**Enough is being done to protect incarcerated individuals from COVID-19**	
Strongly agree / Agree	Ref
Neutral	0.8 (0.3-2.2)
Disagree / Strongly disagree	1.3 (0.6-2.9)
**I believe that the correctional officers take my health concerns seriously.**	
Strongly agree / Agree	Ref
Neutral	0.8 (0.3-1.9)
Disagree / Strongly disagree	1.1 (0.5-2.5)
**I believe that the jail doctors and nurses take my health concerns seriously.**	
Strongly agree / Agree	Ref
Neutral	2.1 (1.0-4.5)*
Disagree / Strongly disagree	2.1 (1.0-4.5)
**I believe that my doctor outside of jail takes my health concerns seriously.**	
Strongly agree / Agree	Ref
Neutral	0.9 (0.5-1.8)
Disagree / Strongly disagree	1.4 (0.6-3.3)
**While in jail/prison, I worry that I will be denied the treatment or services that I need.**	
Strongly agree / Agree	Ref
Neutral	1.3 (0.7-2.4)
Disagree / Strongly disagree	0.6 (0.3-1.4)
**While outside of jail/prison, I worry that I will be denied the treatment or services that I need.**	
Strongly agree / Agree	Ref
Neutral	0.9 (0.4-2.0)
Disagree / Strongly disagree	1.1 (0.6-2.1)

Table S6. Impacts of the COVID-19 pandemic on court dates and routine health care of incarcerated participants. Percentages were calculated after excluding those with missing responses and may not sum up to 100 due to rounding.

	**Percent of respondents**
**Has COVID-19 affected your court date(s)?**	
Yes	61.4
No	34.1
Prefer not to answer	4.5
**In which ways have your court dates been impacted?** (Among participants who selected "Yes" to the above question) (Select all that apply)	
My court date was delayed	75.9
My family/community members could not attend my court date	55.7
My court date was canceled	38.6
Other people (i.e. witnesses who could support my case) were not able to attend	31.6
I could not schedule a court date	14.3
None of the above	2.3
Prefer not to answer	1.1
**About how long has your court date been delayed?** (Among participants who said their court date was delayed)	
Less than 2 weeks	6.2
2-4 weeks	15.8
1-2 months	24.9
Over 2 months	43.8
Indefinitely/I don't know	8.2
Prefer not to answer	1.1
**Were you receiving regular *mental* health care/treatment in jail prior to COVID-19?**	
Yes	38.2
No	47.8
Prefer not to answer	13.9
**How has your regular *mental* health care/treatment in jail changed?** (Among those who responded "Yes" to the above question)	
Increased	12.0
No change	48.5
Decreased	29.8
Stopped	9.7
**Were you receiving regular *physical* health care/treatment in jail prior to COVID-19?**	
Yes	43.2
No	44.2
Prefer not to answer	12.6
**How has your regular *physical* health care/treatment in jail changed?** (Among those who responded "Yes" to the above question)	
Increased	11.2
No change	49.4
Decreased	33.7
Stopped	5.6
